# Aging Does Not Affect Axon Initial Segment Structure and Somatic Localization of Tau Protein in Hippocampal Neurons of Fischer 344 Rats

**DOI:** 10.1523/ENEURO.0043-17.2017

**Published:** 2017-07-21

**Authors:** Andrew Kneynsberg, Nicholas M. Kanaan

**Affiliations:** 1 Neuroscience Program, Michigan State University, East Lansing, MI 48824; 2 Department of Translational Science and Molecular Medicine College of Human Medicine, Michigan State University, Grand Rapids, MI 49503; 3 Hauenstein Neuroscience Center, Mercy Health Saint Mary’s, Grand Rapids, MI 49503

**Keywords:** aging, Alzheimer’s disease, ankyrin G, axon initial segment, Fischer 344, tau

## Abstract

Little is known about the specific contributions of aging to the neuron dysfunction and death in Alzheimer’s disease (AD). AD is characterized by the pathological accumulation of abnormal tau (a microtubule-associated protein), and the mislocalization of tau from the axon to the somatodendritic compartment is thought to play an important role in disease pathogenesis. The axon initial segment (AIS) is thought to play a role in the selective localization of tau in the axonal compartment. Thus, disruption in the AIS barrier may allow tau to diffuse freely back into the somatodendritic compartment and potentially lead to neurotoxicity. Here, we analyzed AISs using stereological methods and protein immunoblotting, and the localization of tau was assessed with immunofluorescence optical density measurements and protein immunoblotting. None of the outcome measurements assessed, including AIS structure, AIS protein levels, the distribution of tau in neurons of the hippocampus (HP), and total tau or phospho-tau protein levels were different in young, middle-, and old-age Fischer 344 rats. The outcome measurements assessed, including AIS structure, AIS protein levels, the distribution of tau in neurons of the HP, and total tau or phospho-tau protein levels were not different in young, middle-, and old-age Fischer 344 rats, with the exception of a small reduction in AIS volume and diameter in the CA2 region of aged animals. These data suggest that aging largely has no effect on these properties of the AIS or tau distribution, and thus, may not contribute directly to tau mislocalization.

## Significance Statement

Aging is a primary risk-factor for AD, but the events during aging that contribute to the development of disease are unknown. In healthy neurons, the tau protein is enriched in the axon, a distribution facilitated, at least in part, by the axon initial segment (AIS). Tau accumulation in the cell body due to disruption of the AIS barrier is potentially a contributing factor to AD pathogenesis. Very little is known about the effects of normal aging on AIS proteins or levels and localization of tau. Here, we report that there were no changes in the AIS or tau localization with increasing age in rats, suggesting disease-specific effects may drive changes in these factors during AD pathogenesis.

## Introduction

Alzheimer’s disease (AD) is the most prevalent neurodegenerative disease of individuals over the age of 65, and affects over 6.4 million adults in the United States (James et al., 2014). The leading risk factor for developing the disease is aging, and while the cause of AD is still unclear, the accumulation of inclusions comprised of tau protein is a hallmark of the disease (Santuccione et al., 2013). It remains unclear whether changes in the distribution of tau that are reminiscent of AD-related changes, such as accumulation in the somatodendritic compartment, occur during normal aging. Aged Fischer 344 rats are commonly used for aging research and exhibit age-related behavioral deficits of learning and memory as well as motor impairment (Spangler et al., 1994; van der Staay and Blokland, 1996; Cochran et al., 2014). Additionally, aging causes altered neuronal protein expression, axonal atrophy, and some gliosis in the F344 rat model (Parhad et al., 1995). For example, glial fibrillary acidic protein (GFAP) is shown to increase significantly in the hippocampus (HP) of aged rats both with and without cognitive impairment (VanGuilder et al., 2011). Thus, Fischer 344 rats are a good model to recapitulate factors of human aging (van der Staay and Blokland, 1996) and determining whether aging effects the AIS and tau distribution. The underlying hypothesis is that aging causes alterations in AIS integrity and tau mislocalization, which may represent events that contribute to aging as a strong risk factor for AD.

Tau, a microtubule-associated protein, is thought to contribute to the development and progression of AD pathology, but the mechanisms behind tau toxicity remain largely unknown. Age-related accumulations of tau were reported in some nonhuman primate brains (Oikawa et al., 2010; Perez et al., 2013), yet systematic analysis of the cellular localization of tau accumulation across aging is not well studied. Unfortunately, human tissue studies are often limited in scope across age groups making it hard to appreciate changes from young to old age. In healthy neurons, tau is enriched in the axonal compartment, where it may stabilize microtubules and play a role in regulating axonal functions such as transport (Tytell et al., 1984; Binder et al., 1986; Kanaan et al., 2013). The redistribution of tau from the axonal compartment to the somatodendritic compartment is thought of as an important event in tau-mediated neurotoxicity (Buee et al., 2000). The preferential localization of tau in axons is mediated, at least in part, by the axon initial segment (AIS), which acts as a retrograde barrier for freely diffusing tau (Li et al., 2011; Sun et al., 2014; Zempel and Mandelkow, 2014; Sohn et al., 2016).

Importantly, very little is known about the connections between aging, the AIS, and tau distribution. The AIS is a selective diffusion barrier separating the axonal compartment from the somatodendritic compartment (Winckler et al., 1999). The establishment of the AIS creates a filter that regulates intracellular traffic based on protein size or the type of motor proteins carrying cargo along microtubules (Song et al., 2009; Leterrier and Dargent, 2014). Ankyrin G (AnkG) is well established as a necessary protein for the development and maintenance of the AIS as a selective filter, as well as a necessary protein for developing and maintaining axonal polarity (Zhou et al., 1998; Hedstrom et al., 2008). AnkG also has an integral role in recruiting and binding structural proteins to the AIS, such as βIV-spectrin and neurofascin (Hedstrom et al., 2007; Freal et al., 2016). Interestingly, a single previous report showed that some structural proteins in the AIS (specifically ankyrin) are reduced with age in wild-type mice (Bahr et al., 1994). Thus, we set out to determine if the structure of the AIS and/or tau localization is altered during normal aging. Our data show that AIS structure, levels of multiple AIS proteins, and the distribution of tau in HP neurons are not altered with advancing age in Fischer 344 rats.

## Materials and Methods

### Animals

Young adult (4 months), middle-aged (14 months) and aged (24 months) male Fischer 344 rats were used for all experiments. Six animals per age group (*n* = 6) were used to obtain fixed tissue for histologic analysis while five or six animals (*n* = 5**-**6) per age group were used for fresh brain homogenate. The animals were provided rat chow and water *ad libitum* and housed in a reverse light-dark cycle room. All animal studies were performed in accordance with standard regulations and were approved by the Michigan State University Institutional Animal Care and Use and Committee.

### Tissue processing

Animals used for collection of fresh brain tissue were transcardially perfused with 200 ml of 0.9% saline containing heparin (10,000 U/l). The brains were extracted and the HP was dissected and frozen on dry ice. For collection of fresh tissue for AnkG immunoblotting, animals were perfused for 5 min with saline containing heparin (∼60 mL). The extracted HPs were then immediately frozen in liquid nitrogen and stored in liquid nitrogen until processing for AnkG immunoblotting. To collect fixed tissue, the saline perfusion was followed by 200 ml phosphate buffered 4% paraformaldehyde. The brains were postfixed in 4% paraformaldehyde for 24 h. After postfixation, the brains were embedded into gelatin blocks for sectioning (Smiley and Bleiwas, 2012). The gelatin block was equilibrated in 20% glycerol. The gelatin block was cut into 40-µm-thick coronal sections on a freezing, sliding stage microtome. Sections were stored in cyroprotectant until processed for immunohistochemistry or immunofluorescence.

### Immunoblotting

Tissue from the HP was homogenized in 300 µl of 10 mM Tris, 1 mM EDTA, 0.8 mM NaCl, and 10% sucrose buffer containing protease and phosphatase inhibitors (10 µg/ml pepstatin, 10 µg/ml leupeptin, 10 µg/ml bestatin, 10 µg/ml aprotinin, 1 mM PMSF, 10 mM β-glycerophosphate, 1 mM sodium orthovanadate, 10 mM sodium fluoride, and 1 mM tetra-sodium pyrophosphate decahydrate), using a sonicator (XL-2000, Misonix, 10 × 1 s bursts at power level 1). Lysates were cleared of cellular debris by centrifugation at 22,000 × *g* for 20 min at 4°C. The resulting supernatants were collected for analysis and the total protein content was assessed using the Bradford protein assay (B6916, Sigma). The samples were diluted in Laemmli buffer and heated to 95°C for 10 min. Lysate samples separated using SDS-PAGE (50 µg total protein/lane for AIS proteins; 20 µg/lane for tau protein) on 4-20% Criterion TGX (Bio-Rad) gradient gels at 250 V and transferred to nitrocellulose membranes for 50 min (66458; Pall Life Sciences) to quantify the amount of AIS proteins and tau between age groups (*n* = 5/group).

Because of the large size of the AnkG protein, a blotting protocol optimized for large molecular weight proteins was used (Fairbanks et al., 1971; Davis and Bennett, 1982; Bolt and Mahoney, 1997). The flash frozen HPs were dropped into nine volumes of urea buffer (8 M urea, 5% SDS, 5 mM N-ethyl maleimide, 10 mM HEPES, 10 μg/ml leupeptin, and 10 μg/ml pepstatin) at 65°C and immediately pulverized with an Eppendorf tube pestle (#12-141-363, Thermo Fisher) for 10 s and then sonicated, as described above. The samples were then diluted in Laemmli buffer and heated to 95°C for 10 min. The protein concentrations of the resulting lysate samples were measured using the SDS Lowry protein assay as described (Cox et al., 2016). Lysate samples (100 μg/lane; *n* = 6/group) were separated using SDS-PAGE on 3-8% Criterion XT Tris-Acetate in XT buffer (Bio-Rad) gradient gels at 150 V and transferred to nitrocellulose membranes in transfer buffer [40 mM Tris, 20 mM sodium acetate, 2 mM EDTA, pH 7.4, 20% (v/v) methanol, 0.05% (w/v) SDS] for 120 min at 10 V. After transferring, the blots were stained with Ponceau S [0.04% (w/v) Ponceau S (#P3504, Sigma), 0.1% (v/v) acetic acid] for 5 min followed by 2 × 5 min washes in 5% acetic acid (v/v) and then two washes in water. The blots were imaged before proceeding to blocking and Ponceau bands were used to normalize the quantified AnkG signals.

**Figure 1.**
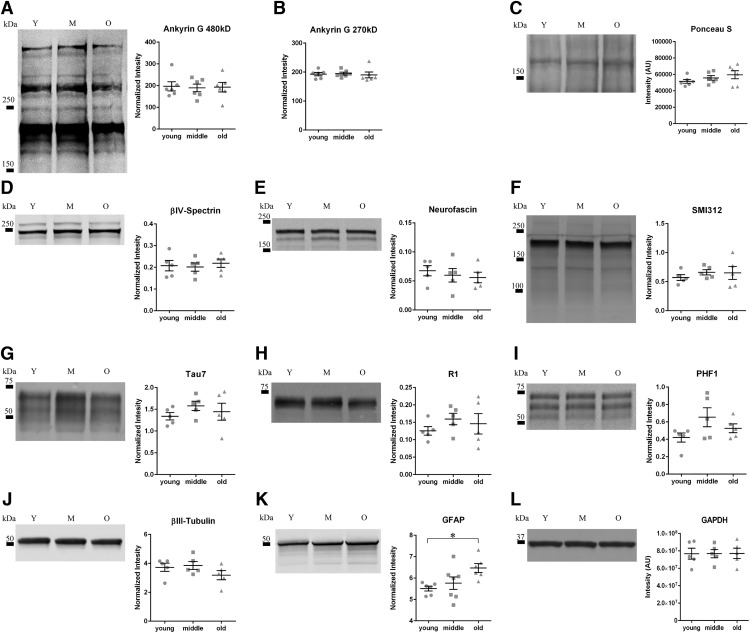
Aging does not alter levels of AIS proteins, total tau, and phosphorylated tau proteins. ***A***, ***B***, Amount of 480-kDa (*A*) and 270-kDa (*B*) isoforms of AnkG do not change between young, middle, and old rats. Note that the other isoforms of AnkG not analyzed are isoforms that are not AIS-specific. ***C***, AnkG Western blottings were normalized to Ponceau S (***C***) staining for loading control. ***D***, ***E***, The amount of βIV-spectrin (***D***) and neurofascin (***E***) also remain unchanged during aging in rats. ***F***, The level of axonal neurofilaments, as indicated by the SMI-312 antibody. The SMI-312 antibody is an axonal marker that labels phospho-neurofilaments in axons. SMI-312 levels are not changed with age, confirming that total axon content is similar across age groups. ***G***, ***H***, The total level of tau detected with Tau7 (***G***) and polyclonal R1 (***H***) do not change with age in the rat HP. ***I***, The levels of PHF1, a phospho-epitope of tau, are not changed with age. ***J***, The total levels of microtubules, labeled with βIII-tubulin, remained unchanged with advancing age. ***K***, GFAP, an astrocytic marker known to increase with age, is significantly increased in the HP of old age rats compared to young and middle aged rats; one-way ANOVA, Tukey’s *post hoc*, *p < 0.05. ***L***, Western blot band intensities are normalized to GAPDH, which did not change with age. All data are displayed as mean ± SEM.

All membranes were blocked in 2% nonfat dry milk in Tris-buffered saline (NFDM-TBS; Tris 50 mM and NaCl 150 mM, pH 7.4) for 1 h at room temperature and incubated with primary antibody in NFDM-TBS overnight at 4°C. Blots were probed with AnkG antibody (H-215, Santa Cruz sc-28561, 1:2000), βIV-spectrin (NeuroMab #75-377, 1:1000), neurofascin (NeuroMab #75-172 1:1000), SMI312 (abcam, ab24574, 1:20,000), R1 (1:100,000; Berry et al., 2004), Tau7 (1:500,000; Horowitz et al., 2006), AT8 (phospho-Ser199/Ser202/Thr205, 1:10,000), PHF-1 (phospho-Ser396/Ser404, 1:50,000), phospho-Ser422 (1:2000), βIII-tubulin antibody (Tuj1, 1:10,000; Caccamo et al., 1989), GFAP (G3893 Sigma, 1:2000), and loading control glyceraldehyde 3-phosphate dehydrogenase (GAPDH; Cell Signaling, 5174, 1:2000). After incubation with primary antibodies, the membranes were washed in TBS and 0.1% Tween 20 and incubated in appropriate species-specific IRDye 680RD or 800CW secondary antibodies (1:20,000 in NFDM-TBS; LI-COR Biotechnology). The membranes were washed and the reactivity visualized with a LI-COR Odyssey infrared imager. The signal intensity for each band was quantified using the LI-COR Image Studio software (v5.2) and signal intensities for AIS proteins or tau are expressed as a ratio to GAPDH signal intensities. AnkG 270 and 480 kDa are expressed as a ratio of the Ponceau S intensity of a protein band at ∼200 kDa. The intensities of GAPDH and Ponceau that were used as loading controls for normalization of immunoblotting signals were not changed across age [GAPDH, *F*_(2,12)_ = 0.001261, *p* = 0.9987 (Fig. 1*L*); Ponceau S, *F*_(2,15)_ = 1.322, *p* = 0.2959 (Fig. 1*C*)].

### Immunohistochemistry and immunofluorescence

A one in six series of tissue sections were processed for each age group (*n* = 6) for immunohistochemical detection of AnkG. The tissue was rinsed in 0.1 M TBS, pH 7.4, containing 0.5% Triton X-100 (TBS-Tx) six times for 10 min each. The tissue was then incubated in 3% H_2_O_2_ in TBS-Tx for 1 h at room temperature to quench endogenous peroxidase activity, and then rinsed again. An avidin/biotin blocking kit (Vector Labs SP-2001) was used to block endogenous avidin in the gelatin matrix. Nonspecific antibody binding was inhibited by incubating the tissue in blocking buffer [10% goat serum (GS)/2% bovine serum albumin/0.5% TBS-Tx] for 1 h at room temperature. The tissue was then incubated over night at 4°C with rabbit anti-AnkG primary antibody (H-215, Santa Cruz sc-28561) 1:1000 in dilution buffer (2%GS and 0.5% TBS-Tx). The tissue was rinsed (6 × 10 min in 0.5% TBS-Tx) and incubated for 2 h in biotinylated goat anti-rabbit secondary antibody (BA-1000, Vector) at a concentration of 1:500 in dilution buffer. The tissue was then rinsed again and incubated in avidin-biotin complex (ABC) solution (PK-6100, Vector) for 1hour. The ABC solution contained 50 µl of solution A and 50 µl of solution B in 10 ml of TBS made according to the manufacturer’s instructions. After rinsing the tissue, the staining was developed using 3,3'-diaminobenzidine (D5637, Sigma) solution (50 mg/ml, 0.5% TBS-Tx, and 0.003% H_2_O_2_) for 12 min. The tissue was then rinsed, mounted on microscope slides, and coverslipped with CYTOSEAL 60 (#8310-16, Thermo Scientific).

A one in six series of tissue sections (*n* = 6/group) was used for the immunofluorescent labeling of tau. All tissue was stained simultaneously in staining dishes using the same reagents. Nonspecific antibody binding was inhibited by incubating the tissue in blocking buffer (10% GS, 2% bovine serum albumin, and 0.5% TBS-Tx) for 1 h at room temperature. The tissue was then incubated over night at 4°C with mouse anti-tau primary antibody (Tau7; 1:3500) in dilution buffer (2%GS and 0.5% TBS-Tx). The tissue was rinsed (6 × 10 min in 0.5% TBS-Tx) and incubated for 2 h in Alexa Fluor 488-conjugated goat anti-mouse IgG (H + L; #A-11001, Thermo Fisher) at a concentration of 1:500 in dilution buffer. The tissue was then incubated in DAPI (0.5 µg/ml in 0.5% TBS-Tx for 10 min), rinsed (5 × 10 min in 0.5% TBS-Tx), and the sections were mounted on microscope slides. To block endogenous autofluorescence, the tissue was treated with Sudan black. The slides were incubated in 70% ethanol for 2 min, then in a saturated solution of Sudan Black B (Fisher, #AC419830100) for 5 min. The slides were then differentiated in 70% ethanol until background (gray matter) was pale gray, and rinsed in dH_2_O (2 × 3 min). The slides were then coverslipped using Vectashield hardset mountant (H-1400, Vector; Kanaan et al., 2008).

### Stereology

The spaceballs stereological probe is an unbiased and systematic stereological sampling method to estimate the total length of a population of fibers in 3D space, and this probe was used to quantify AIS length in the HP of rats. This stereological method was performed using serial sections (one in six series). Sampling grids (i.e., CA: 500 × 500 µm; DG: 200 × 500 µm) were chosen for each region to allow for ∼200 fiber intersections to be counted in the HP for each hemisphere and to yield a Gunderson coefficient of error <0.1 for all samples. The mean coefficient of error was 0.058 ± 0.001 (SEM). A hemisphere probe with a radius of 11 µm was used to sample sites throughout the HP. The mean measured tissue thickness was ∼13-14 µm. A 4× objective was used to outline each region and a 60× oil objective lens (1.35 numerical aperture) was used for all stereological counts.

The AutoNeuron probe in Neurolucida (MBF Bioscience) was used to acquire measurements of individual AIS length, volume and diameter in the regions of the HP (CA1, CA2, CA3 and dentate gyrus) in young, middle, and old aged animals. In each region, an image stack was acquired with a 60× oil objective lens through the complete depth of each section at a step-size of 0.5 µm. A 3D representation of each individual AIS in the HP was created in the software using the following settings for AutoNeuron: image type, brightfield; max process diameter, 2.00 µm; XY region, all; Z range, all; trace somas, no; soma sensitivity, 50; min soma diameter, 2.00 µm; seed detection sensitivity, 70; seed response filter, 4; tracing sensitivity, 60; tolerance to gaps, low; and connect branches, no. These settings were chosen to best detect the AIS of each neuron, while not falsely detecting background tissue stain or failing to differentiate individual AISs.

### Cellular protein quantification

The tau immunofluorescence-stained tissue was imaged on a Nikon AI confocal system and a Nikon Eclipse Ti microscope with a 40× oil objective (1.30 numerical aperture). Image stacks (0.5 µm step size) were acquired of neurons in each HP region (CA1, CA2, CA3, and the dentate gyrus) using acquisition settings in the linear range of fluorescent intensity without saturation of tau signal and the same acquisition settings were used for all animals. Four image stacks were acquired per animal. The neurons were analyzed with NIS Elements (v4.30, Advanced Research, Nikon). The average intensity of Tau7 immunofluorescent signal was measured in the soma of individual pyramidal neurons in all CA regions (CA1, CA2, and CA3). Cells were chosen as randomly as possible, but only cells with an entire cross-section through the middle of the cell (i.e., the z-slice with the largest nuclear width) within the acquired z-stack and without overlapping cell bodies were used for analysis. To increase the rigor of this analysis, the experimenter was blinded to the condition of the samples. A box (∼8-12 µm^2^) was drawn as large as possible inside the somatic compartment of each neuron, excluding the DAPI-positive nuclei. The mean value of fluorescence intensity was measured for each box within a total of 180 cells/animal/group. The density and small cytoplasm of dentate granule cells precludes the ability to reliably measure signal intensity within individual cells. Instead, the average fluorescence intensity within a rectangular box (∼5000 µm^2^; six boxes/animal/group) was used to perform a regional analysis of immunofluorescence signal intensity within the dentate gyrus (Fig. 3*F-I*).

For axonal tau intensity measurements, a 4x objective (0.13 numerical aperture) was used to capture two to three images of the dorsal HP in three serial tissue sections per animal. The entire visible regions of the fimbria, alveus, stratum lacunosum, and stratum moleculare were outlined in NIS Elements software. The average fluorescent intensity per area was measured for each axonal region.

### Statistical analyses

All data were analyzed using Prism software (v6.0) and all data are presented as mean ± SEM. Statistical significance between age groups was determined using one-way ANOVA. Significance was set at *p* < 0.05. Tukey’s *post hoc* test was used for *post hoc* comparisons when significance of *p* < 0.05 was reached. If no overall significance was achieved, no *post hoc* analyses were used.

## Results

### Immunoblotting of AIS proteins and tau

Immunoblots of young, middle, and old age tissue were probed for AIS protein markers, including AnkG, βIV-spectrin, and neurofascin. When normalized to GAPDH loading controls, no changes were detected between aged groups (βIV-spectrin *F*_(2,12)_ = 0.1095, *p* = 0.8972; neurofascin *F*_(2,12)_ = 0.3654, *p* = 0.7014; Fig. 1*D*,*E*). AnkG protein bands were analyzed at 480 and 270 kDa for the AIS-localized AnkG isoforms [*F*_(2,15)_ = 0.03397, *p* = 0.9667 (Fig. 1*A*); *F*_(2,15)_ = 0.08969, *p* = 0.9147 (Fig. 1*B*); Kordeli et al., 1995; Zhang and Bennett, 1998]. Additional AnkG protein bands were detected at ∼190 kDa that did not change with age (data not shown), but these represent other canonical isoforms that are not specific to the AIS (Bennett and Baines, 2001). Blots were probed with SMI-312, an axon-specific neurofilament antibody, to confirm that aging-related axonal loss was not present in the HP. No change in the amount of SMI-312 was detected across age groups (*F*_(2,12)_ = 0.4304, *p* = 0.6599; Fig. 1*F*).

There was no change across age groups in the levels of total tau protein as indicated by Tau7, a C-terminal pan-tau antibody (*F*_(2,12)_ = 0.7686, *p* = 0.4852; Fig. 1*G*). As a secondary method to confirm there were no changes in total tau levels we also probed blots with the R1 tau antibody, a pan-tau rabbit polyclonal antibody (*F*_(2,12)_ = 0.6824, *p* = 0.5240; Fig. 1*H*) and found no aging-related changes (Berry et al., 2004; Horowitz et al., 2006). To determine if specific tau phosphor epitopes were changed with aging in the HP we probed blots with PHF1 antibody and found no differences (*F*_(2,12)_ = 2.361, *p* = 0.1366; Fig.1*I*). We also probed with the AT8 antibody and the phospho-Ser422 antibody, both of which are disease-related modifications of tau (Guillozet-Bongaarts et al., 2006; Jeganathan et al., 2008) and found no signal for either epitope in young, middle or old age animals (data not shown). Tubulin was analyzed to measure the amount of microtubules as a control since tau is a microtubule-associated protein. There was no change in βIII-tubulin across age groups (*F*_(2,12)_ = 0.8679, *p* = 0.4446; Fig. 1*J*).

As a positive control for detection of age-related changes of protein, blots were probed for GFAP, a protein previously shown to change in HP lysates of aging rats (VanGuilder et al., 2011). A significant increase in GFAP was found in the old rats compared with the young rats (*F*_(2,16)_ = 4.529, *p* = 0.0276; Fig. 1*K*).

### Stereological assessment of AIS length and morphology

**Figure 2.**
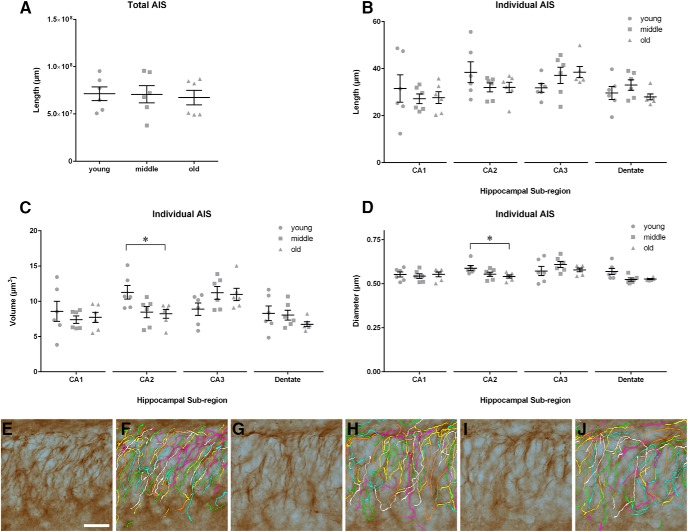
Structural analysis of the AIS with AnkG reveals regional changes in the HP during aging. ***A***, The total length of AISs in the HP estimated with the spaceballs stereology probe is unchanged across age groups. ***B-D***, The Neurolucida AutoNeuron analysis reveals that the length (***B***), volume (***C***), and diameter (***D***) of the AISs across young, middle, and old aged rats is not changed in CA1, CA3, or the dentate gyrus of the HP. The volume and diameter of the AISs in the CA2 region were significantly reduced in the old age compared with the young rats (one-way ANOVA, Tukey’s *post hoc*, *p < 0.05). All data are displayed as mean ± SEM. ***E-G***, Representative images of the AISs (AnkG-positive immunostain, brown) in the dentate gyrus of the HP at ages 4 (***E***), 14 (***G***), and 24 (***I***) months, and the corresponding Neurolucida AutoNeuron tracings (***F***, ***H***, and ***J***; multicolor overlay to visually differentiate individual AISs). Scale bar, 20 µm (***E-I***).

Stereological analysis of the AIS length (using the spaceballs probe in AnkG-stained tissue) showed no differences in the total length of all AISs in the HP across age groups (*F*_(2,15)_ = 0.07323, *p* = 0.9297; Fig. 2*A*). We used the AutoNeuron module as a second method to analyze individual AISs in the CA1, CA2, CA3, and dentate gyrus regions of the HP and found that length did not change across age groups in any of these regions (length: CA1: *F*_(2,15)_ = 0.3940, *p* = 0.6811; CA2: *F*_(2,15)_ = 1.488, *p* = 0.2573; CA3: *F*_(2,15)_ = 1.770, *p* = 0.2041; DG: *F*_(2,15)_ = 1.413, *p* = 0.2739; Fig. 2*B*). No aging-related change occurred in the volume or diameter of individual AISs in CA1, CA3, or DG (volume: CA1: *F*_(2,15)_ = 0.3936, *p* = 0.6814; CA3: *F*_(2,15)_ = 2.077, *p* = 0.1599; DG: *F*_(2,15)_ = 1.216, *p* = 0.3241 and diameter: CA1: *F*_(2,15)_ = 0.1559, *p* = 0.8545; CA3: *F*_(2,15)_ = 1.061, *p* = 0.3706; DG: *F*_(2,15)_ = 0.6705, *p* = 0.5261; Fig. 2*C*,*D*). However, a small but significant change was detected in the volume and diameter of CA2 neurons when comparing young and old animals (volume: CA2: *F*_(2,15)_ = 4.460, *p* = 0.0302; and diameter: CA2: *F*_(2,15)_ = 4.062, *p* = 0.0389; Fig. 2*C*,*D*). The measured length of the AIS in HP neurons was 32.86 ± 1.99 µm in young, 32.33 ± 1.379 µm in middle, and 31.53 ± 1.361 µm in old aged animals (Fig. 2*B*), consistent with previous findings in cultured HP neurons (Grubb and Burrone, 2010; Leterrier et al., 2015).

### Optical density measurements of tau levels in somata and axonal layers of the HP

**Figure 3.**
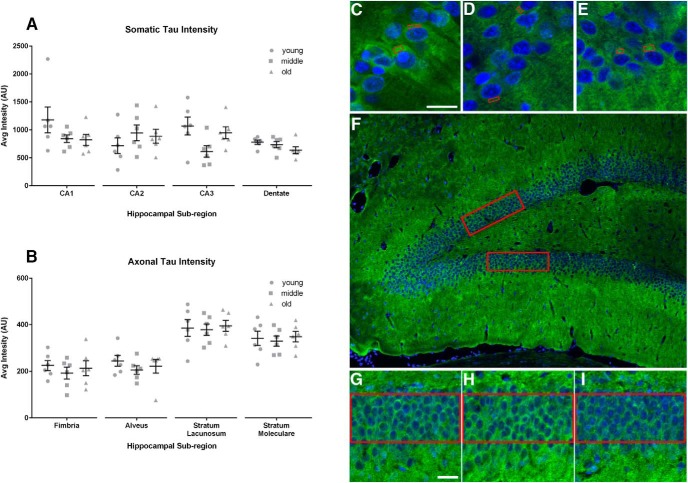
Optical density measurement of tau immunofluorescence in the somata and axons of HP neurons shows no changes across age. ***A***, The intensity of somatic tau (using Tau7, a total tau antibody) in neurons in the CA1, CA2, CA3, and dentate regions of the HP shows no change with advancing age. ***B***, No change in the regional intensity of tau is detected in the axon-enriched strata of the HP (i.e., the fimbria, alveus, stratum lacunosum, and stratum moleculare). All data are displayed as mean ± SEM. ***C-E***, Representative images of CA1 HP neurons positive for Tau7 at 4 (***C***), 14 (***D***), and 24 (***E***) months. The red rectangles are examples of the areas that fluorescence intensities were measured within individual neurons. ***F***, Image illustrating the regions (in red) used for analysis of somatic tau intensity in the dentate gyrus. ***G-I***, Enlargements represent the analyzed regions of 4-month-old (***G***), 14-month-old (***H***), and 24-month-old (***I***) rats. Scale bar, 20 µm.

Optical density measurements of Tau7 immunofluorescence in the somatic compartment of individual HP neurons in tissue sections showed no change across age groups (Fig. 3*A*). Individual analysis of pyramidal neurons in the CA regions did not show an age-related difference (CA1: *F*_(2,15)_ = 1.775, *p* = 0.2033; CA2: *F*_(2,15)_ = 0.7810, *p* = 0.4757; CA3: *F*_(2,15)_ = 3.513, *p* = 0.0561; Fig. 3*A*), nor did the dentate gyrus show a change in regional intensity with advancing age in rats (*F*_(2,15)_ = 1.924, *p* = 0.1804; Fig. 3*A*). Analysis of tau intensity in HP strata enriched in axonal projections did not detect any change with age (fimbria: *F*_(2,15)_ = 0.3902, *p* = 0.6836; alveus: *F*_(2,15)_ = 0.6694, *p* = 0.5267; stratum lacunosum: *F*_(2,15)_ = 0.08560, *p* = 0.9184; stratum moleculare: *F*_(2,15)_ = 0.1433, *p* = 0.8676; Fig. 3*B*).

## Discussion

With the etiology of AD still unknown, it is important to investigate known risk factors and pathologic changes associated with the disease. The axonal enrichment of tau may deteriorate during the pathogenesis of AD as tau appears to accumulate in the somatodendritic compartment. Considering that aging is the leading risk factor for AD, investigation into the normal aging process may lead to the discovery of previously unappreciated anomalous features that contribute to disease vulnerability. Thus, we investigated the integrity of the AIS, the barrier involved in maintaining axonal localization of tau, over the span of aging in rats to establish whether normal aging might affect the AIS structure and/or tau distribution.

We evaluated age-related changes in tau and the AIS using multiple markers and complementary approaches. However, we found no evidence that the levels of AIS proteins or tau proteins change with age in the HP, nor levels of somatic or axonal tau change in HP neurons with advancing age in rats. The length of the AIS was not found to change in any region of the HP, but a discrete and specific effect was detected in the CA2 region (∼8% decrease from young to old). The reduction of AIS volume and diameter without a change in length indicates possible atrophy the axonal projections specifically in CA2 neurons, but biological significance of these changes in the context of tau distribution and AIS functionality remain unclear. Overall, the findings presented here provide a strong case against aging-related changes in the total tau levels, somatic tau levels, some phosphorylated forms of tau (i.e., PHF1, AT8, pS422), levels of AIS structural proteins (i.e., AnkG, neurofascin, βIV-spectrin), or AIS morphology within the HP, thus, these factors are unlikely to contribute to the risk of developing AD. Importantly, these findings do not rule out that other variables related to tau and/or the AIS (e.g., other forms of tau, other characteristics/functions of the AIS, etc.) are changed during normal aging and might contribute to susceptibility for AD.

Before this work, a single study assessed the aging changes in the AIS and showed changes in ankyrin and spectrin proteins using Western blottings of telencephalic tissue from aged mice (Bahr et al., 1994). The discordant findings reported here could be due to the use of rats instead of mice, the methods used to immunoblot ankyrin or the increased specificity of antibodies currently available to the isoforms of proteins localized to the AIS (i.e., ankyrin vs AnkG 480/270 kDa or spectrin vs βIV-spectrin).

Multiple complimentary experimental approaches were used in the evaluation of the effects of aging on both tau and the AIS. Examination of aging-related changes in total tau protein levels was conducted through Western blotting using multiple tau markers, however, these methods do not allow cell-specific measurements. To assess aging-related changes specifically within levels of somatic tau we used fluorescence intensity measurements within the soma of individual HP neurons. Since tau is a microtubule-associated protein, we wanted to establish whether changes in tubulin content was altered with advancing age, but there were no age-related changes in total tubulin levels and the amount of tau per tubulin remained constant within the HP, which aligns well with previous studies showing HP neurons are not lost in normal aging (both in humans and in rats; West et al., 2004; Stanley et al., 2012). These findings demonstrate that the total amount of tau and the amount of tau in the soma remain unchanged in the HP of aging rats.

The same level of rigor was applied to the investigation of the AIS by using a multifaceted approach to evaluate changes in aging rats. Multiple key AIS proteins (AnkG, βIV-spectrin, and neurofascin) were examined in Western blottings, and we measured SMI312 levels to determine if total axonal content was changed. The lack of aging changes in all AIS proteins assessed and SMI312 suggest that axonal content and key structural components of the AIS are not altered with age. This conclusion is further supported by the lack of changes in tubulin described above. Stereological analysis of the AIS was conducted using two complementary approaches. The space balls probe is an established methodology for detecting aging-related changes in fiber structures within the brain (Ypsilanti et al., 2008), and here we used it to measure AIS length. The AutoNeuron method in Neurolucida was used to perform 3D measurements of individual AISs and showed a lack of age-related changes in AIS length and only a mild, region-specific decrease in width and volume. This probe has been used previously to measure the diameter of dendrites in pyramidal neurons and compare them across brain regions (Amatrudo et al., 2012).The unbiased stereological measurement of AIS length in the entire HP is a significant advantage of this approach to analyze AIS morphology. The length of the AIS was previously measured with βIV-spectrin using a nonstereological method in selected z-stacks of the HP (Baalman et al., 2013). Interestingly, they report that a shortening of AISs correlates to cognitive impairment following explosion-induced brain trauma in Sprague Dawley rats. This work suggests that a shortening of the AIS may correlate to cognitive impairment, but we did not detect a shortening of AISs with age in the current work. Collectively, these data strongly demonstrate that the gross structure of the AIS (i.e., length, diameter, volume) do not change during the normal aging process in most cells of the HP.

Although we are reporting mostly negative data, it is unlikely that the Fischer 344 rat was not an appropriate model of aging to use for this study. We included age groups that span the spectrum from young (4 months), middle (14 months), and old (24 months), which is near the end of the normal lifespan and a time at which aging-related impairments in memory and cognition occur. For example, an age-related decline in learning ability at 24 months of age was observed using a 14-unit T-maze and shock-motivated one-way active avoidance test in Fischer 344 rats (Spangler et al., 1994). Additionally, 24-month-old Fischer 344 rats exhibit pathologies in multiple organ systems associated with advanced aging, further supporting their utility as a model of aging. We confirmed that our techniques and methods are capable of detecting other changes in normal aging that were previously reported (i.e., GFAP increase; VanGuilder et al., 2011).

Several studies showed that there is heterogeneity among aged animals on a number of learning and memory functions (e.g., water maze), as well as other behavioral tasks, indicating that individual animals respond to the aging process differently (Gage et al., 1984; Collier and Coleman, 1991; Freeman et al., 2009). These studies, made it clear that aged animals can be separated into unimpaired or impaired groups based on performance and that these changes correspond to some neurochemical and neuroanatomical changes. However, the data reported here demonstrate that two distinct populations of aged animals do not exist in regard to the specific AIS and tau parameters studied, suggesting these variables do not underlie the behavioral and cognitive decline seen in aging Fischer 344 rats. Analysis of these parameters in young, middle, and old humans may be necessary to definitively establish whether the same holds true for humans.

The negative findings presented here provide important insight into the aging-related changes in tau and AIS in the HP. This information is important considering the contention in the field that tau mislocalization is important in AD and the recent focus on the role of the AIS in tau distribution (Zempel et al., 2010; Li et al., 2011; Sohn et al., 2016). Thus, future investigations should focus on alternative aspects of tau and the AIS to further identify whether aging-related changes may contribute to the risk of AD.

## Synthesis

Reviewing Editor: Vivian Budnik, University of Massachusetts Decisions are customarily a result of the Reviewing Editor and the peer reviewers coming together and discussing their recommendations until a consensus is reached. When revisions are invited, a fact-based synthesis statement explaining their decision and outlining what is needed to prepare a revision will be listed below. The following reviewer(s) agreed to reveal their identity: Matthew Grubb, Ying Xu.

The following items should be addressed before the manuscript is deemed suitable for publication in eNeuro. Please see reviewer's comments (appended at the end) for further details on these requirements.

**Data analysis**

1. Authors should present AIS and Tau data separately for each individual hippocampal region, including images and analyses for each (see comments from reviewer 1 for details).

2. Clarify the statistical analysis performed in the GFAP immunoblot.

**Clarifications/corrections**

1. Provide information about the Tau secondary antibody and the parameters for sudan black used

2. Provide details about sample preparation and image quantification for Tau

3. Correct labeling errors in Figure 2 and those highlighted by Reviewer 2

**Experimental**

1. Are the levels of AnkG 480kDa isoform constant during aging?

2. Besides measuring Tau levels in the soma, the authors should conduct these measurements in the axonal compartment

**Discussion**

1. Discuss findings by Baalman et al (2012)

2. Discuss the interpretation of results along the lines of Reviewer 2 (comment 1 )

**Reviewers comments**

**Reviewer 1**

This is a generally well-conducted and well-presented manuscript describing negative results that are nonetheless both important and interesting. The authors use a combination of immunoblotting and histological techniques to demonstrate that normal ageing is not associated with large alterations in the composition or morphology of the axon initial segment (AIS), nor with large changes in somatic tau. Before publishing in eNeuro, I have one major and several minor comments that can be readily addressed. Doing so would considerably increase my confidence in the results reported here.

Major comment:

The data presented in Figures 2 and 3 would be much stronger if they were presented separately for all individual regions of the hippocampus (i.e. CA1 vs CA3 vs DG). AIS length is known to vary significantly across different hippocampal projection neuron subtypes (e.g. Evans et al. 2013, 2015), so including all regions in one combined analysis has the potential to mask important differences between age groups. Please provide example images and separate analyses (or, better, a combined 2-way ANOVA with region and age as factors) for different hippocampal subregions, both for the AIS and the tau analyses (for the latter, DG is currently analysed separately, but CA1-3 are not).

Minor comments

- Recent data have shown that the giant 480kDa isoform of AnkG is most important for AIS formation. Can the authors report whether abundance of this isoform is also constant with ageing?

- In the methods, please provide details of the secondary antibody used for tau immunofluorescence, as well as the sudan black concentration/incubation time/supplier.

- Please also provide details of the imaging for tau immunofluorescence quantification - i.e. was detection in the linear, non-saturating range, and were all images obtained in the same session, or at least with the same settings? Were slices from young/middle/old animals stained together in the same well, or at least using the same batch of reagents?

- Please clarify the results of the GFAP immunoblot analysis. The p value in the main text refers to the 1-way ANOVA, but the figure legend reports the same p value for a post-test. Which post-tests were performed - is the old group different from both other groups?

- In Figure 2, the plot labels need to say ‘individual’. And EFGHI are mistakenly labelled.

- The discussion mentions ‘To our knowledge, this is the first stereological measurement of AIS length in tissue’, but something very similar was carried out by Baalman et al. 2012 J Neurotrauma

**Reviewer 2**

The present study investigated whether aging affects the AIS and tau distribution in Fischer 344 rats. The author found that axon initial segment (AIS) structure, AIS protein levels, the distribution of tau in neurons of the hippocampus, and total tau or phospho-tau protein levels were not different among young, middle-, and old-age groups. From these data, the author made a conclusion that aging-related changes in these specific variables may not contribute directly to tau mislocalization. This is an interesting paper. But there are some major concerns on the experimental designs.

1. AD is an aging related disease, however, not all people get AD when they are aged. In the same respect, although Fischer 344 rats at 24 months of age exhibited age-related decline in learning ability, it doesn't mean that all the rats at 24 months lose their memory. It would be much more convincible if the authors evaluated the learning ability of two rats, i.e. abnormal aging with memory deficits and normal aging without memory deficits, and analyzed correlation of learning ability and AIS protein levels or tau distribution between them.

2. In the introduction, the author mentioned ‘the redistribution of tau from the axonal compartment to the somatodendritic compartment is thought of as an important event in tau-mediated neurotoxicity’. However, in the present study, the author just analyzed somatic tau intensity in the dentate gyrus. It would be better if the tau distribution was also evaluated from axonal compartment.

Minor comments

1. In the discussion, the author mentioned that ‘We included age groups that span the spectrum from young (mths), middle (mths) and old age (24 mths),...’. The author missed some numbers before ‘mths’.

2. In Fig 2G-I, what dose different color line mean?

3. In Fig 3, figure G-I was not from figure F. So please delete the black line.

## References

[B1] Amatrudo JM, Weaver CM, Crimins JL, Hof PR, Rosene DL, Luebke JI (2012) Influence of highly distinctive structural properties on the excitability of pyramidal neurons in monkey visual and prefrontal cortices. J Neurosci 32:13644–13660. 10.1523/JNEUROSCI.2581-12.2012 23035077 PMC3485081

[B2] Baalman KL, Cotton RJ, Rasband SN, Rasband MN (2013) Blast wave exposure impairs memory and decreases axon initial segment length. J Neurotrauma 30:741–751. 10.1089/neu.2012.2478 23025758 PMC3941920

[B3] Bahr BA, Lam N, Lynch G (1994) Changes in the concentrations of tau and other structural proteins in the brains of aged mice. Neurosci Lett 175:49–52. 10.1016/0304-3940(94)91075-8 7970209

[B4] Bennett V, Baines AJ (2001) Spectrin and ankyrin-based pathways: metazoan inventions for integrating cells into tissues. Physiol Rev 81:1353–1392. 11427698 10.1152/physrev.2001.81.3.1353

[B5] Berry JD, Jones S, Drebot MA, Andonov A, Sabara M, Yuan XY, Weingartl H, Fernando L, Marszal P, Gren J, Nicolas B, Andonova M, Ranada F, Gubbins MJ, Ball TB, Kitching P, Li Y, Kabani A, Plummer F (2004) Development and characterisation of neutralising monoclonal antibody to the SARS-coronavirus. J Virol Methods 120:87–96. 10.1016/j.jviromet.2004.04.009 15234813 PMC7119589

[B6] Binder LI, Frankfurter A, Rebhun LI (1986) Differential localization of MAP-2 and tau in mammalian neurons in situ. Ann NY Acad Sci 466:145–166. 3089105 10.1111/j.1749-6632.1986.tb38392.x

[B7] Bolt MW, Mahoney PA (1997) High-efficiency blotting of proteins of diverse sizes following sodium dodecyl sulfate-polyacrylamide gel electrophoresis. Anal Biochem 247:185–192. 10.1006/abio.1997.2061 9177676

[B8] Buee L, Bussiere T, Buee-Scherrer V, Delacourte A, Hof PR (2000) Tau protein isoforms, phosphorylation and role in neurodegenerative disorders. Brain Res Brain Res Rev 33:95–130. 10967355 10.1016/s0165-0173(00)00019-9

[B9] Caccamo D, Katsetos CD, Herman MM, Frankfurter A, Collins VP, Rubinstein LJ (1989) Immunohistochemistry of a spontaneous murine ovarian teratoma with neuroepithelial differentiation. Neuron-associated beta-tubulin as a marker for primitive neuroepithelium. Lab Invest 60:390–398. 2467076

[B10] Cochran JN, Hall AM, Roberson ED (2014) The dendritic hypothesis for Alzheimer's disease pathophysiology. Brain Res Bull 103:18–28. 10.1016/j.brainresbull.2013.12.004 24333192 PMC3989444

[B11] Collier TJ, Coleman PD (1991) Divergence of biological and chronological aging: evidence from rodent studies. Neurobiol Aging 12:685–693. 10.1016/0197-4580(91)90122-Z 1791906

[B12] Cox K, Combs B, Abdelmesih B, Morfini G, Brady ST, Kanaan NM (2016) Analysis of isoform-specific tau aggregates suggests a common toxic mechanism involving similar pathological conformations and axonal transport inhibition. Neurobiol Aging 47:113–126. 10.1016/j.neurobiolaging.2016.07.015 27574109 PMC5075521

[B13] Davis J, Bennett V (1982) Microtubule-associated protein 2, a microtubule-associated protein from brain, is immunologically related to the alpha subunit of erythrocyte spectrin. J Biol Chem 257:5816–5820. 6175631

[B14] Fairbanks G, Steck TL, Wallach DF (1971) Electrophoretic analysis of the major polypeptides of the human erythrocyte membrane. Biochemistry 10:2606–2617. 10.1021/bi00789a030 4326772

[B15] Freal A, Fassier C, Le Bras B, Bullier E, De Gois S, Hazan J, Hoogenraad CC, Couraud F (2016) Cooperative interactions between 480 kDa ankyrin-G and EB proteins assemble the axon initial segment. J Neurosci 36:4421–4433. 10.1523/JNEUROSCI.3219-15.2016 27098687 PMC6601828

[B16] Freeman WM, VanGuilder HD, Bennett C, Sonntag WE (2009) Cognitive performance and age-related changes in the hippocampal proteome. Neuroscience 159:183–195. 10.1016/j.neuroscience.2008.12.004 19135133 PMC2701617

[B17] Gage FH, Dunnett SB, Björklund A (1984) Spatial learning and motor deficits in aged rats. Neurobiol Aging 5:43–48. 6738785 10.1016/0197-4580(84)90084-8

[B18] Grubb MS, Burrone J (2010) Activity-dependent relocation of the axon initial segment fine-tunes neuronal excitability. Nature 465:1070–1074. 10.1038/nature09160 20543823 PMC3196626

[B19] Guillozet-Bongaarts AL, Cahill ME, Cryns VL, Reynolds MR, Berry RW, Binder LI (2006) Pseudophosphorylation of tau at serine 422 inhibits caspase cleavage: in vitro evidence and implications for tangle formation in vivo. J Neurochem 97:1005–1014. 10.1111/j.1471-4159.2006.03784.x 16606369

[B20] Hedstrom KL, Ogawa Y, Rasband MN (2008) AnkyrinG is required for maintenance of the axon initial segment and neuronal polarity. J Cell Biol 183:635–640. 10.1083/jcb.200806112 19001126 PMC2582894

[B21] Hedstrom KL, Xu X, Ogawa Y, Frischknecht R, Seidenbecher CI, Shrager P, Rasband MN (2007) Neurofascin assembles a specialized extracellular matrix at the axon initial segment. J Cell Biol 178:875–886. 10.1083/jcb.200705119 17709431 PMC2064550

[B22] Horowitz PM, LaPointe N, Guillozet-Bongaarts AL, Berry RW, Binder LI (2006) N-terminal fragments of tau inhibit full-length tau polymerization in vitro. Biochemistry 45:12859–12866. 10.1021/bi061325g 17042504

[B23] James BD, Leurgans SE, Hebert LE, Scherr PA, Yaffe K, Bennett DA (2014) Contribution of Alzheimer disease to mortality in the United States. Neurology 82:1045–1050. 10.1212/WNL.0000000000000240 24598707 PMC3962992

[B24] Jeganathan S, Hascher A, Chinnathambi S, Biernat J, Mandelkow EM, Mandelkow E (2008) Proline-directed pseudo-phosphorylation at AT8 and PHF1 epitopes induces a compaction of the paperclip folding of tau and generates a pathological (MC-1) conformation. J Biol Chem 283:32066–32076. 10.1074/jbc.M805300200 18725412

[B25] Kanaan NM, Kordower JH, Collier TJ (2008) Age-related changes in dopamine transporters and accumulation of 3-nitrotyrosine in rhesus monkey midbrain dopamine neurons: relevance in selective neuronal vulnerability to degeneration. Eur J Neurosci 27:3205–3215. 10.1111/j.1460-9568.2008.06307.x 18598263 PMC3391583

[B26] Kanaan NM, Pigino GF, Brady ST, Lazarov O, Binder LI, Morfini GA (2013) Axonal degeneration in Alzheimer's disease: when signaling abnormalities meet the axonal transport system. Exp Neurol 246:44–53. 10.1016/j.expneurol.2012.06.003 22721767 PMC3465504

[B27] Kordeli E, Lambert S, Bennett V (1995) AnkyrinG. A new ankyrin gene with neural-specific isoforms localized at the axonal initial segment and node of Ranvier. J Biol Chem 270:2352–2359. 10.1074/jbc.270.5.2352 7836469

[B28] Leterrier C, Dargent B (2014) No Pasaran! Role of the axon initial segment in the regulation of protein transport and the maintenance of axonal identity. Semin Cell Dev Biol 27:44-51. 10.1016/j.semcdb.2013.11.001 24239676

[B29] Leterrier C, Potier J, Caillol G, Debarnot C, Rueda Boroni F, Dargent B (2015) Nanoscale architecture of the axon initial segment reveals an organized and robust scaffold. Cell Rep 13:2781–2793. 10.1016/j.celrep.2015.11.051 26711344

[B30] Li X, Kumar Y, Zempel H, Mandelkow EM, Biernat J, Mandelkow E (2011) Novel diffusion barrier for axonal retention of tau in neurons and its failure in neurodegeneration. EMBO J 30:4825–4837. 10.1038/emboj.2011.376 22009197 PMC3243615

[B31] Oikawa N, Kimura N, Yanagisawa K (2010) Alzheimer-type tau pathology in advanced aged nonhuman primate brains harboring substantial amyloid deposition. Brain Res 1315:137–149. 10.1016/j.brainresrev.2009.12.005 20004650

[B32] Parhad IM, Scott JN, Cellars LA, Bains JS, Krekoski CA, Clark AW (1995) Axonal atrophy in aging is associated with a decline in neurofilament gene expression. J Neurosci Res 41:355–366. 10.1002/jnr.490410308 7563228

[B33] Perez SE, Raghanti MA, Hof PR, Kramer L, Ikonomovic MD, Lacor PN, Erwin JM, Sherwood CC, Mufson EJ (2013) Alzheimer's disease pathology in the neocortex and hippocampus of the western lowland gorilla (*Gorilla gorilla gorilla*). J Comp Neur 521:4318–4338. 10.1002/cne.23428 23881733 PMC6317365

[B34] Santuccione AC, Merlini M, Shetty A, Tackenberg C, Bali J, Ferretti MT, McAfoose J, Kulic L, Bernreuther C, Welt T, Grimm J, Glatzel M, Rajendran L, Hock C, Nitsch RM (2013) Active vaccination with ankyrin G reduces β-amyloid pathology in APP transgenic mice. Mol Psychiatry 18:358–368. 10.1038/mp.2012.70 22688190

[B35] Smiley JF, Bleiwas C (2012) Embedding matrix for simultaneous processing of multiple histological samples. J Neurosci Methods 209:195–198. 10.1016/j.jneumeth.2012.06.005 22710286 PMC3619222

[B36] Sohn PD, Tracy TE, Son HI, Zhou Y, Leite RE, Miller BL, Seeley WW, Grinberg LT, Gan L (2016) Acetylated tau destabilizes the cytoskeleton in the axon initial segment and is mislocalized to the somatodendritic compartment. Mol Neurodegener 11:47. 10.1186/s13024-016-0109-0 27356871 PMC4928318

[B37] Song AH, Wang D, Chen G, Li Y, Luo J, Duan S, Poo MM (2009) A selective filter for cytoplasmic transport at the axon initial segment. Cell 136:1148–1160. 10.1016/j.cell.2009.01.016 19268344

[B38] Spangler EL, Waggie KS, Hengemihle J, Roberts D, Hess B, Ingram DK (1994) Behavioral-assessment of aging in male Fischer-344 and brown-Norway rat strains and their F(1) hybrid. Neurobiol Aging 15:319–328. 10.1016/0197-4580(94)90027-2 7936056

[B39] Stanley EM, Fadel JR, Mott DD (2012) Interneuron loss reduces dendritic inhibition and GABA release in hippocampus of aged rats. Neurobiol Aging 33:431.e1–e13. 10.1016/j.neurobiolaging.2010.12.014 PMC3110542 21277654

[B40] Sun X, Wu Y, Gu M, Liu Z, Ma Y, Li J, Zhang Y (2014) Selective filtering defect at the axon initial segment in Alzheimer's disease mouse models. Proc Natl Acad Sci USA 111:14271–14276. 10.1073/pnas.1411837111 25232037 PMC4191768

[B41] Tytell M, Brady ST, Lasek RJ (1984) Axonal transport of a subclass of tau proteins: evidence for the regional differentiation of microtubules in neurons. Proc Natl Acad Sci USA 81:1570–1574. 10.1073/pnas.81.5.1570 6200879 PMC344879

[B42] van der Staay FJ, Blokland A (1996) Behavioral differences between outbred Wistar, inbred Fischer 344, brown Norway, and hybrid Fischer 344 x brown Norway rats. Physiol Behav 60:97–109. 8804648 10.1016/0031-9384(95)02274-0

[B43] VanGuilder HD, Bixler GV, Brucklacher RM, Farley JA, Yan H, Warrington JP, Sonntag WE, Freeman WM (2011) Concurrent hippocampal induction of MHC II pathway components and glial activation with advanced aging is not correlated with cognitive impairment. J Neuroinflammation 8:138. 10.1186/1742-2094-8-138 21989322 PMC3216278

[B44] West MJ, Kawas CH, Stewart WF, Rudow GL, Troncoso JC (2004) Hippocampal neurons in pre-clinical Alzheimer's disease. Neurobiol Aging 25:1205–1212. 10.1016/j.neurobiolaging.2003.12.005 15312966

[B45] Winckler B, Forscher P, Mellman I (1999) A diffusion barrier maintains distribution of membrane proteins in polarized neurons. Nature 397:698–701. 10.1038/17806 10067893

[B46] Ypsilanti AR, Girão da Cruz MT, Burgess A, Aubert I (2008) The length of hippocampal cholinergic fibers is reduced in the aging brain. Neurobiol Aging 29:1666–1679. 10.1016/j.neurobiolaging.2007.04.001 17507114

[B47] Zempel H, Mandelkow E (2014) Lost after translation: missorting of tau protein and consequences for Alzheimer disease. Trends Neurosci 37:721–732. 10.1016/j.tins.2014.08.004 25223701

[B48] Zempel H, Thies E, Mandelkow E, Mandelkow EM (2010) Abeta oligomers cause localized Ca(2+) elevation, missorting of endogenous tau into dendrites, tau phosphorylation, and destruction of microtubules and spines. J Neurosci 30:11938–11950. 10.1523/JNEUROSCI.2357-10.2010 20826658 PMC6633549

[B49] Zhang X, Bennett V (1998) Restriction of 480/270-kD ankyrin(G) to axon proximal segments requires multiple ankyrin(G)-specific domains. J Cell Biol 142:1571–1581. 10.1083/jcb.142.6.1571 9744885 PMC2141775

[B50] Zhou DX, Lambert S, Malen PL, Carpenter S, Boland LM, Bennett V (1998) Ankyrin(G) is required for clustering of voltage-gated Na channels at axon initial segments and for normal action potential firing. J Cell Biol 143:1295–1304. 10.1083/jcb.143.5.1295 9832557 PMC2133082

